# Supplementary material for: Shades of yellow: interactive effects of visual and odour cues in a pest beetle
Source: PeerJ. 2016 Jul 12;4:e2219. doi: 10.7717/peerj.2219 (PMC4950555; doi:10.7717/peerj.2219)
Supplement: Supplemental Information 2 — a) Results of Rayleigh test analysis (z-scores) of S. zeamais orientation vectors and b) binomial test probabilities (Bonferroni-corrected to a significance value of 0.0016) in the presence and absence of host odours. Visual stimuli were presented at 90° to the camera azimuth and odour stimuli at 180° to the camera azimuth. (In a), * indicates p < 0.05, ** p < 0.01, *** p < 0.001; in b) # indicates significance after correction.). [file peerj-04-2219-s002.docx]

**A**

**Model design:**

Intercept + Treatment + Sex + Age + Starv + Treatment * Sex + Treatment * Age + Treatment * Starv + Sex * Age + Sex * Starv + Age * Starv + Treatment * Sex * Age + Treatment * Sex * Starv + Treatment * Age * Starv + Sex * Age * Starv + Treatment * Sex * Age * Starv

Where “Starv” is the period of food deprivation

**B**

| **Effect** | **Value** | ***F*** | **Hypothesis df** | **Error df** | ***p*-value** |
| --- | --- | --- | --- | --- | --- |
| Intercept | .356 | 1.782b | 5.000 | 25.000 | .153 |
| Treatment | 2.724 | 2.124 | 30.000 | 117.000 | .002 |
| Sex | .077 | .385b | 5.000 | 25.000 | .854 |
| Age | 5.780 | 1.288 | 105.000 | 117.000 | .091 |
| Period of food deprivation | 3.377 | 2.258 | 35.000 | 117.000 | .001 |
| Treatment * Sex | .415 | 2.073b | 5.000 | 25.000 | .103 |
| Treatment * Age | .228 | 1.141b | 5.000 | 25.000 | .365 |
| Treatment * Period of food deprivation | .000 | .b | .000 | 2.000 | . |
| Sex * Age | 1.068 | .999 | 25.000 | 117.000 | .474 |
| Sex * Period of food deprivation | .000 | .b | .000 | 2.000 | . |
| Age * Period of food deprivation | .000 | .b | .000 | 2.000 | . |
| Treatment * Sex * Age | .000 | .b | .000 | 2.000 | . |
| Treatment * Sex * Period of food deprivation | .000 | .b | .000 | 2.000 | . |
| Treatment * Age * Period of food deprivation | .000 | .b | .000 | 2.000 | . |
| Sex * Age * Period of food deprivation | .000 | .b | .000 | 2.000 | . |
| Treatment * Sex * Age * Period of food deprivation | .000 | .b | .000 | 2.000 | . |

| **Source** | **Recording period** | **Type III Sum of Squares** | **df** | **Mean Square** | ***F*** | ***p*-value** |
| --- | --- | --- | --- | --- | --- | --- |
| Corrected Model | P1 | 2870588.684a | 86 | 33378.938 | 1.678 | .058 |
|  | P4 | 5209959.817d | 86 | 60580.928 | 1.223 | .275 |
| Intercept | P1 | 646.826 | 1 | 646.826 | .033 | .858 |
|  | P4 | 216459.546 | 1 | 216459.546 | 4.371 | .045 |
| Treatment | P1 | 473732.575 | 6 | 78955.429 | 3.969 | .005 |
|  | P4 | 218772.401 | 6 | 36462.067 | .736 | .625 |
| Sex | P1 | 11191.776 | 1 | 11191.776 | .563 | .459 |
|  | P4 | 55057.259 | 1 | 55057.259 | 1.112 | .300 |
| Age | P1 | 656711.864 | 21 | 31271.994 | 1.572 | .128 |
|  | P4 | 454057.445 | 21 | 21621.783 | .437 | .973 |
| Period of food deprivation | P1 | 422895.831 | 7 | 60413.690 | 3.037 | .016 |
|  | P4 | 724672.927 | 7 | 103524.704 | 2.091 | .077 |
| Treatment * Sex | P1 | 4382.440 | 1 | 4382.440 | .220 | .642 |
|  | P4 | 97281.610 | 1 | 97281.610 | 1.965 | .172 |
| Treatment * Age | P1 | 55855.808 | 1 | 55855.808 | 2.807 | .105 |
|  | P4 | 5292.000 | 1 | 5292.000 | .107 | .746 |
| Treatment * Period of food deprivation | P1 | .000 | 0 | . | . | . |
|  | P4 | .000 | 0 | . | . | . |
| Sex * Age | P1 | 81738.101 | 5 | 16347.620 | .822 | .544 |
|  | P4 | 294876.107 | 5 | 58975.221 | 1.191 | .338 |
| Sex * Period of food deprivation | P1 | .000 | 0 | . | . | . |
|  | P4 | .000 | 0 | . | . | . |
| Age * Period of food deprivation | P1 | .000 | 0 | . | . | . |
|  | P4 | .000 | 0 | . | . | . |
| Treatment * Sex * Age | P1 | .000 | 0 | . | . | . |
|  | P4 | .000 | 0 | . | . | . |
| Treatment * Sex * Period of food deprivation | P1 | .000 | 0 | . | . | . |
|  | P4 | .000 | 0 | . | . | . |
| Treatment * Age * Period of food deprivation | P1 | .000 | 0 | . | . | . |
|  | P4 | .000 | 0 | . | . | . |
| Sex * Age * Period of food deprivation | P1 | .000 | 0 | . | . | . |
|  | P4 | .000 | 0 | . | . | . |
| Treatment * Sex * Age * Period of food deprivation | P1 | .000 | 0 | . | . | . |
|  | P4 | .000 | 0 | . | . | . |
| Error | P1 | 576970.148 | 29 | 19895.522 |  |  |
|  | P4 | 1436058.109 | 29 | 49519.245 |  |  |
