# Supplementary material for: Shades of yellow: interactive effects of visual and odour cues in a pest beetle
Source: PeerJ. 2016 Jul 12;4:e2219. doi: 10.7717/peerj.2219 (PMC4950555; doi:10.7717/peerj.2219)
Supplement: Supplemental Information 3 [file peerj-04-2219-s003.docx]

**A**

| Colour | *N* | *z* without odour | *z* with odour |
| --- | --- | --- | --- |
| Amber | 15 | 2.169624* | 2.01539* |
| Orange | 15 | 0.533501 | 1.595403 |
| Yellow | 31 | 6.673843*** | 10.2351*** |
| Yellow (textured) | 16 | 2.077826* | 0.155136 |
| Sand | 16 | 2.702122* | 1.064188 |
| Black | 19 | 14.98742*** | 9.486128*** |
| Grating | 19 | 3.419676*** | 3.399831*** |
| Control | 17 | 0.986605 | 1.816808 |

**B**

| **Colour** | **Period 1, visual direction** | **Period 4, visual direction** | **Period 4, odour direction** | **Period 4, intermediate visual/odour** |
| --- | --- | --- | --- | --- |
| Amber | 0.1134 | 0.01908 | 0.92212 | 0.25847 |
| Orange | 0.23153 | 0.9351 | 0.0274 | 1 |
| Yellow | 0.00282 | 0.22348 | 0.00054# | 0.00022# |
| Yellow (textured) | 0.23153 | 0.77284 | 0.51322 | 0.36981 |
| Sand | 0.9351 | 0.70404 | 0.42054 | 0.11167 |
| Black | <0.0001# | <0.0001# | 0.74039 | 0.14837 |
| Grating | 0.02808 | 0.31129 | 0.31129 | 0.42611 |
| Control | 0.77284 | 0.27091 | 0.27091 | 0.80289 |
